# Retinal alpha-synuclein accumulation correlates with retinal dysfunction and structural thinning in the A53T mouse model of Parkinson’s disease

**DOI:** 10.3389/fnins.2023.1146979

**Published:** 2023-05-05

**Authors:** Katie K. N. Tran, Vickie H. Y. Wong, Anh Hoang, David I. Finkelstein, Bang V. Bui, Christine T. O. Nguyen

**Affiliations:** ^1^Department of Optometry and Vision Sciences, The University of Melbourne, Parkville, VIC, Australia; ^2^The Florey Institute of Neuroscience and Mental Health, The University of Melbourne, Parkville, VIC, Australia

**Keywords:** A53T, alpha-synuclein, Parkinson’s disease, retina, electroretinography, optical coherence tomography

## Abstract

Abnormal alpha-synuclein (α-SYN) protein deposition has long been recognized as one of the pathological hallmarks of Parkinson’s disease’s (PD). This study considers the potential utility of PD retinal biomarkers by investigating retinal changes in a well characterized PD model of α-SYN overexpression and how these correspond to the presence of retinal α-SYN. Transgenic A53T homozygous (HOM) mice overexpressing human α-SYN and wildtype (WT) control littermates were assessed at 4, 6, and 14  months of age (male and female, *n* = 15–29 per group). *In vivo* retinal function (electroretinography, ERG) and structure (optical coherence tomography, OCT) were recorded, and retinal immunohistochemistry and western blot assays were performed to examine retinal α-SYN and tyrosine hydroxylase. Compared to WT controls, A53T mice exhibited reduced light-adapted (cone photoreceptor and bipolar cell amplitude, *p* < 0.0001) ERG responses and outer retinal thinning (outer plexiform layer, outer nuclear layer, *p* < 0.0001) which correlated with elevated levels of α-SYN. These retinal signatures provide a high throughput means to study α-SYN induced neurodegeneration and may be useful *in vivo* endpoints for PD drug discovery.

## 1. Introduction

Parkinson’s disease (PD) is a neurodegenerative disorder that although is well-known for its motor manifestations, also affects other sensory systems (Postuma et al., 2019). There remains no clinical test that can definitively diagnose PD, and clinical diagnosis of PD can take up to 5 years (Adler et al., 2014). The accessible nature of the retina through the clear optics of the eye, and its shared embryological origins with the brain (Sinn and Wittbrodt, 2013), provides the potential means for PD-related neuronal changes to be monitored (Miri et al., 2016; Hu et al., 2022). Visual symptoms such as poor visual acuity (Jones et al., 1992; Matsui et al., 2006; Lin et al., 2015), contrast sensitivity (Bulens et al., 1986; Bodis-Wollner et al., 1987; Mestre et al., 1990) and color vision (Büttner et al., 1995; Oh et al., 2011; Sun et al., 2014) as well as findings of decreased retinal function as indicated by delays and attenuations in both outer and inner retinal components of both photopic and scotopic electroretinograms (Nightingale et al., 1986; Tartaglione et al., 1987; Peppe et al., 1995; Tagliati et al., 1996; Nowacka et al., 2015; Mello et al., 2022) have been established in PD patients. Associated with these functional deficits meta-analyses across many studies (20–36 papers) show structural thinning at the fovea and inner retinal layers however the outer retina is less well studied (Huang et al., 2020, 2021; Zhou et al., 2021). Whether these changes are specifically driven by PD-related pathological processes remain unclear.

A key pathology of PD is alpha-synuclein (α-SYN) rich Lewy bodies (LBs) which are abnormal cytoplasmic intraneuronal protein deposits found in post-mortem brains of PD patients (Hughes et al., 1992). Several studies have established the presence of LBs and phosphorylated α-SYN in the inner retina of post-mortem PD samples (Beach et al., 2014; Bodis-Wollner et al., 2014; Veys et al., 2019), the levels of which correlate with PD duration and disease severity (Ortuño-Lizarán et al., 2018). Chorostecki et al. (2015) propose that accumulation of retinal α-SYN may correspond to the increase in outer plexiform layer volume they found in individuals living with PD. Whether the accumulation of α-SYN in specific retinal layers can account for structural and functional changes is not well understood. In this regard, animal models of α-SYN deposition may provide insight into this protein’s role in instigating PD-related changes in retinal structure and function.

In the Thy-1 A30P mouse (mutation in A30P synuclein alpha, *SNCA*), Veys et al. (2021) observed human and phosphorylated (pSer129) α-SYN immunoreactivity in the ganglion cell layer (GCL) and inner plexiform layer (IPL), which coincided with IPL thinning from 12 months of age and a delay in ganglion cell timing (positive scotopic threshold response, pSTR) at 18 months of age. In the A53T mouse (mutation in A53T synuclein alpha, *SNCA*), Mammadova et al. (2019) observed progressive human and pSer129 α-SYN accumulation in the outer nuclear layer (ONL) at 5 months of age that spread further through the retina with advancing age (8 and 12–18 months old), or with inoculation from brain homogenates. The current study examines whether retinal α-SYN deposition in the A53T mouse model relates to retinal thickness and function. By assessing a range of ages (4, 6, 14 months old) that span early to advanced PD (Oaks et al., 2013; Billings et al., 2016) a time-course of progression will be assessed. This approach will also return a spread of data that will facilitate correlation of α-SYN to retinal structure and function, enabling insight into the underlying factors contributing to *in vivo* measurements that can be done in human patients.

## 2. Materials and methods

### 2.1. General procedures – A53T animals, ethics, and husbandry

All experimental procedures were performed in accordance with the National Health and Medical Research Council Australian Code of Practice for the care and use of animals for scientific purposes and ARRIVE (Animal Research: Reporting of *in vivo* Experiments) guidelines (Percie du Sert et al., 2020). Ethics approval was obtained from The Florey Institute of Neuroscience and Mental Health Animal Ethics Committee (Approval number: 17-046-UM). Mice used in this study were colonized from the A53T transgenic M83 line (JAX stock #004479; B6;C3-Tg(Prnp-SNCA*A53T)83Vle/J); which expresses the autosomal dominant mutant human A53T synuclein-alpha (*SNCA*) gene, under a prion promoter (Giasson et al., 2002). As the original background strain (B6C3H) of these mice expresses the retinal degeneration allele Pde6b^rd1^, all animals were genotyped for the A53T and Pde6b^rd1^ gene (Transnetyx, Cordova, TN, USA) and only mice without the Pde6b^rd1^ gene were included in this study.

In total, a balanced gender mixture of 119 adult female and male A53T homozygous (HOM, *n* = 55) and wildtype (WT, *n* = 64) control mice were bred within the animal facility of the Melbourne Brain Centre (Kenneth Myer Building, Parkville, VIC, Australia). All mice were housed in a well-ventilated environment that was kept at constant room temperature (21°C) and operated through a 12-h diurnal light–dark cycle. To reduce and control ocular photo-oxidative stress, room illumination was kept to <50 lux (Penn and Williams, 1986). Mouse chow (Barastoc, Melbourne, VIC, Australia) and water were provided *ad libitum*.

Animals were assessed cross-sectionally at three ages: 4 (WT, *n* = 16; HOM, *n* = 16), 6 (WT, *n* = 19; HOM, *n* = 15) and 14 months of age (WT, *n* = 29; HOM, *n* = 24). Animals from WT and HOM groups were randomly examined within each testing session where *in vivo* retinal structure (optical coherence tomography, OCT) was assessed first then retinal function (electroretinography, ERG) 1 week later.

Before experimentation, mice were weighed and anesthetized with an intraperitoneal injection (Troy Laboratory, Smithfield, NSW, Australia) of ketamine (80 mg/kg) and xylazine (10 mg/kg) diluted in sterile saline (1:10) to facilitate hydration and administration. Corneal anaesthesia and pupil mydriasis were achieved with 0.5% proxymetacaine and 1% tropicamide drops (Alcaine™ and Mydriacyl™, respectively, Alcon Laboratories, Frenchs Forest, NSW, Australia). Lubricating eye drops or eye gel (Systane® or GenTeal®, respectively, Novartis Pharmaceuticals Australia) was used to maintain ocular hydration. A heat pad was used to maintain body temperature at 37.5 ± 0.5°C throughout *in vivo* assessments. Following *in vivo* assessment mice were perfused with phosphate buffered saline (PBS) solution and eye tissue were collected *post-mortem* for *ex vivo* histological and protein assessment.

### 2.2. Optical coherence tomography

Spectral domain optical coherence tomography (SD-OCT, Spectralis®, Heidelberg Engineering, Heidelberg, Germany) was used to quantify *in vivo* retinal structure (A53T HOM, *n* = 15–24/age; WT, *n* = 16–29/age). Retinal volume scans (8.1 × 8.1 × 1.9 mm) centered on the optic nerve head were acquired, each sampling 121 evenly distributed B-scans, each consisting of 768 A-scans (3.87 μm axial vs. 9.8 μm lateral resolution). OCT scans were collected at an average speed of 85,000 A-scans per second with automated real-time averaging of 6 frames per second.

OCT analysis was undertaken using the Heidelberg Eye Explorer 2 OCT reader plugin (Heyex, Heidelberg Engineering). Automatic segmentation of all retinal layers (retinal nerve fiber layer, RNFL; ganglion cell inner plexiform layer, GCIPL; inner nuclear layer, INL; outer plexiform layer, OPL; outer nuclear layer, ONL) and total retinal thickness (TRT) was enabled by an in-built algorithm within the Heidelberg Eye Explorer 2 software. An annulus [Early Treatment Diabetic Retinopathy Study (ETDRS) outer 6 mm diameter ring] positioned on the optic nerve head was used for analysis as previously conducted (Blades et al., 2020; Tran et al., 2022).

### 2.3. Electroretinography

Mice underwent overnight dark adaptation (12 h) to maximize retinal sensitivity (Behn et al., 2003) before full-field electroretinography (ERG) was performed (A53T HOM, *n* = 9–13/age; WT, *n* = 9–14/age) as previously described (Nguyen et al., 2016; Zhao et al., 2017; Lim et al., 2020; Tran et al., 2022). In brief, ERG recordings constituted dark-adapted and light-adapted responses to target the rod and cone pathways. After anaesthesia and mydriasis, a pair of active and inactive chlorided silver (A&E Metal Merchants, Sydney, NSW, Australia) electrodes connected to platinum leads (F-E-30, Grass Telefactor, West Warwick, RI) were placed on the corneal apex and sclera, respectively. A stainless-steel electrode (F-E2, Grass Telefactor) was inserted subcutaneously into the tail as a ground reference.

To achieve even retinal illumination, a customized Ganzfeld sphere (Photometric Solutions International, Oakleigh, VIC, Australia) delivered calibrated (IL1700, International Light technologies, Peabody, MA) light stimuli of ascending luminous energies. ERG responses were simultaneously measured from both eyes. Scope™ software (Powerlab ADInstruments, Bella Vista, NSW, Australia) was used for signal acquisition which was sampled at a rate of 4 kHz over 640 ms. Band-pass filtering (0.3 to 1,000 Hz, −3 dB) was applied to signals to reduce high frequency noise, which were then digitally saved (ML785 Powerlab 8SP, ADInstruments) for *post hoc* processing.

To probe dark-adapted responses, light stimuli ranging from −5.01 to 2.07 log cd.s/m^2^ was used to generate photoreceptoral (a-wave, P3), bipolar cell (b-wave, P2) and ganglion cell (positive scotopic threshold response, pSTR) driven components (amplitude) of the ERG. To assess the light-adapted response (cone pathway), animals adapted to a 125 cd/m^2^ background for 15 min during which responses were tracked every minute (2.72 log cd.s/m^2^). Upon stabilization, a sequence of stimuli ranging from 0.3 to 2.72 log cd.s/m^2^ was delivered to isolate the a-wave (P3) and b-wave (P2) of the cone pathway.

ERG waveform analysis was undertaken using Excel™ (Microsoft, Redmond, WA, USA) as previously described in detail (Nguyen et al., 2016). In short, a delayed Gaussian function was used to model the a-wave (the first electronegative component of the ERG waveform) to give the P3 (Lamb and Pugh, 1992) over the two brightest luminous energies (rod P3 at 1.55 and 2.07 log cd·s/m^2^ and cone P3 at 2.20 and 2.72 log cd·s/m^2^) from which photoreceptoral amplitude (RmP3, μV) was derived. The P3 was subtracted from the waveform to isolate the P2-OP complex. A discrete Fourier transform and digital filters were applied to the complex to obtain the P2 (b-wave, low pass filter, 46.9 Hz, −3 dB) (Hood and Birch, 1992) mediated by ON-bipolar cells. A saturated hyperbolic function was used to model peak amplitudes of P2 waveforms (Fulton and Rushton, 1978) across all intensities to yield bipolar cell amplitude (Vmax, μV). Finally, retinal ganglion cell function was assessed using pSTR peak amplitude (μV) averaged from two luminous energies −5.01 and − 4.90 log cd.s/m^2^ (Saszik et al., 2002). As the bandpass filters used during data collection were not amenable to accurately measuring temporal data, amplitude information was returned but we conservatively elected not to analyze ERG timings.

### 2.4. Histological and protein assays

After *in vivo* experiments, animals were perfused with 0.1 M phosphate buffered saline (PBS) and eyes were enucleated for western blot or immunohistochemical analysis.

#### 2.4.1. Western blotting

Fresh retinal tissue was dissected from one eye of each animal with the aid of a microscope and snap frozen in liquid nitrogen immediately after collection and stored at −80°C. Retinae were assayed individually (A53T HOM, *n* = 6/age; WT, *n* = 6/age, balanced in gender) then pooled (6 retina of the same age group and genotype) for representative blots. Each sample was homogenized using a probe sonicator (10 s × 3; Branson digital sonifier, Model S450, Danbury, CT, USA) in 100 μl of radioimmunoprecipitation assay (RIPA) lysis buffer [500 mM Tris (pH 7–8.0), 150 mM NaCl, 0.5% sodium dodecyl sulfate (SDS), 1% IGEPAL® CA-630 (Sigma-Aldrich, St. Louis, Missouri, MO, USA), 0.5% sodium deoxycholate (DOC) and protease inhibitor tablet (cOmplete™ Mini Protease Inhibitor Cocktail tablet, Roche Diagnostic, Basel, Switzerland)] then vortexed every 10 min for 1 h while kept on ice. Homogenates were then centrifuged at 15,000 *g* for 15 min at 4°C to separate the protein supernatant from the pellet containing cellular debris. Total protein concentration was determined by a Bradford protein assay (Pierce™ Coomassie Plus, Thermo Fisher Scientific, Waltham, Massachusetts, USA) and aliquots of 50 μg per sample were used for western blots.

Retinal protein samples (50 μg) were mixed in 4X sample buffer (NuPAGE™ LDS [lithium dodecyl sulfate] Sample Buffer, Invitrogen, Thermo Fisher Scientific, Carlsbad, USA) with 5% beta-mercaptoethanol and MilliQ water and heated to 70°C for 10 min. Protein was electrophoresed on 4–12% polyacrylamide gels (NuPAGE™, Bis-Tris, 1.0 mm, Midi Protein Gel, Invitrogen, Thermo Fisher Scientific) at 200 V for 40 min and transferred onto 0.45 μm (pore-size) PVDF membrane (Immobilon-P, Merck, Sigma-Aldrich) at 20 V over 1 h. Membranes were submerged in Ponceau S staining solution (Thermo Fisher Scientific) and measurements of total protein in each lane was captured and quantified using the ChemiDoc MP imaging system (BioRad, Hercules, California, USA). Membranes were then washed and blocked in tris-buffered saline (TBS, Tris 20 mM, NaCl 150 mM, pH 7.6) containing 5% skim milk powder (Woolworths, Australia) overnight at 4°C then incubated at room temperature with primary and secondary antibodies for 1 h at each step. All antibodies (human alpha-synuclein, 1:1000, #ab138501 [MJFR1], Abcam, Cambridge, UK; pSer129 alpha-synuclein 1:1000, #ab209422 [EP1536Y], Abcam; mouse alpha-synuclein, 1:2000, #D37A6, Dako, Glostrup, Denmark; tyrosine hydroxylase (TH), 1:1000, #AB152, Merck, Kenilworth, New Jersey, USA) were diluted in TBS containing 5% skim milk powder. Membranes were washed in tris-buffered saline with 0.01% Tween-20 (TBS-T) for 30 min (6 × 5 min) before and after secondary antibody incubation. Enhanced chemiluminescence (ECL, Clarity Kit, BioRad) was used to detect protein bands which were visualized with the ChemiDoc MP imaging system (BioRad) and quantified by densitometry (ImageLab 6.1, BioRad). The relative protein abundance of each cell lysate was normalized to the respective lane’s automated TP measurement via ChemiDoc stain-free detection software.

#### 2.4.2. Immunohistochemistry

Immunohistochemistry was conducted on retinal tissue from each cohort (A53T HOM, *n* = 3/age; WT, *n* = 3/age) and used for representative comparison. Following enucleation, eyes were fixed in 4% paraformaldehyde (PFA, Sigma-Aldrich, St. Louis, Missouri, USA) diluted in 0.1 M PBS for 1 h at room temperature.

Eyes were then washed with PBS and the anterior segment (cornea, iris, lens) was dissected and removed. The posterior eye cup was cryoprotected using graded sucrose steps (10, 20, and 30%) overnight and then embedded in Tissue Tek Optimal Cutting Temperature (OCT, Sakura Finatek, Torrance, CA) compound which was immediately snap frozen and stored at −20°C. Retinal cross sections (14 μm thick) were cryosectioned in the sagittal plane on a cryostat (Leica, Wetzlar, Germany) and collected onto SuperFrost™ plus glass slides (Invitrogen, Thermo Fisher Scientific, Carlsbad, CA, USA). Slides with retinal tissue surrounding the optic nerve head and that maintained structural integrity at this region of interest post-staining were chosen for immunohistological processing.

After defrosting, slides were washed with PBS to remove OCT compound from sections. Retinae were then incubated for 24 h at 4°C with primary antibodies (human alpha-synuclein, 1:500, #ab138501 [MJFR1], Abcam; pSer129 alpha-synuclein 1:200, #ab209422 [EP1536Y], Abcam; mouse alpha-synuclein, 1:500, #D37A6, Dako; tyrosine hydroxylase [TH], 1:500, #AB152, Merck) diluted in a permeabilization blocking buffer of 0.5% Triton X-100, 5% goat serum and PBS. Following overnight primary antibody incubation, slides were washed and incubated with goat anti-rabbit Alexa-647 (1:500, #A-21244, Invitrogen) diluted in PBS for 2 h at room temperature. Retinal sections were then washed, counter-stained with Hoechst (1:1000, Roche Applied Science, Mannheim, Germany), washed again, mounted with fluorescent mounting medium (DAKO, Glostrup, Denmark) and cover-slipped. Z-stack images (optimal section thickness set to achieve 50% overlap) were acquired at a central region of each cross section using a Zeiss LSM880 confocal laser microscope (Carl Zeiss AG, Oberkochen, Germany) with a 20x air objective lens and Zen Black software (3.0 SR [Black]; Carl Zeiss AG) at 3428 × 3,428 pixel-resolution. Images were then post-processed in FIJI software (National Institutes of Health, Bethesda, MD, USA) to obtain maximum intensity projection then analyzed.

### 2.5. Data and statistical analysis

To evaluate differences between genotype and age effects, statistical comparisons for all group data were conducted using Prism 8 software (GraphPad Software Inc., San Diego, USA) and are expressed as group mean ± standard error of the mean (SEM) unless stated otherwise. In some instances, group data have been normalized and expressed relative to the average of the 4-month WT control cohort or to the 4-month HOM group in cases where WT levels were zero (i.e. Western blot). Outliers were identified with, a ROUT test and removed from analysis. Generally, either a two-tailed unpaired Student’s *t*-test or two-way analysis of variance (ANOVA) with Sidak’s correction for *post hoc* multiple comparisons. In instances of repeated measures analysis with subject drop out (e.g., due to ROUT outlier testing), a generalized liner mixed model analysis was implemented (McGilchrist, 1993; Cnaan et al., 1997). A Deming regression and Spearman’s rank correlation (*R_s_*) were used to determine association between two parameters that had variable x and y axes. An alpha of 0.05 was employed to determine statistical significance.

## 3. Results

### 3.1. Immunohistochemistry and western blot protein assay

#### 3.1.1. Elevated alpha-synuclein levels detected in the retinae of A53T mice

**Figure 1:**
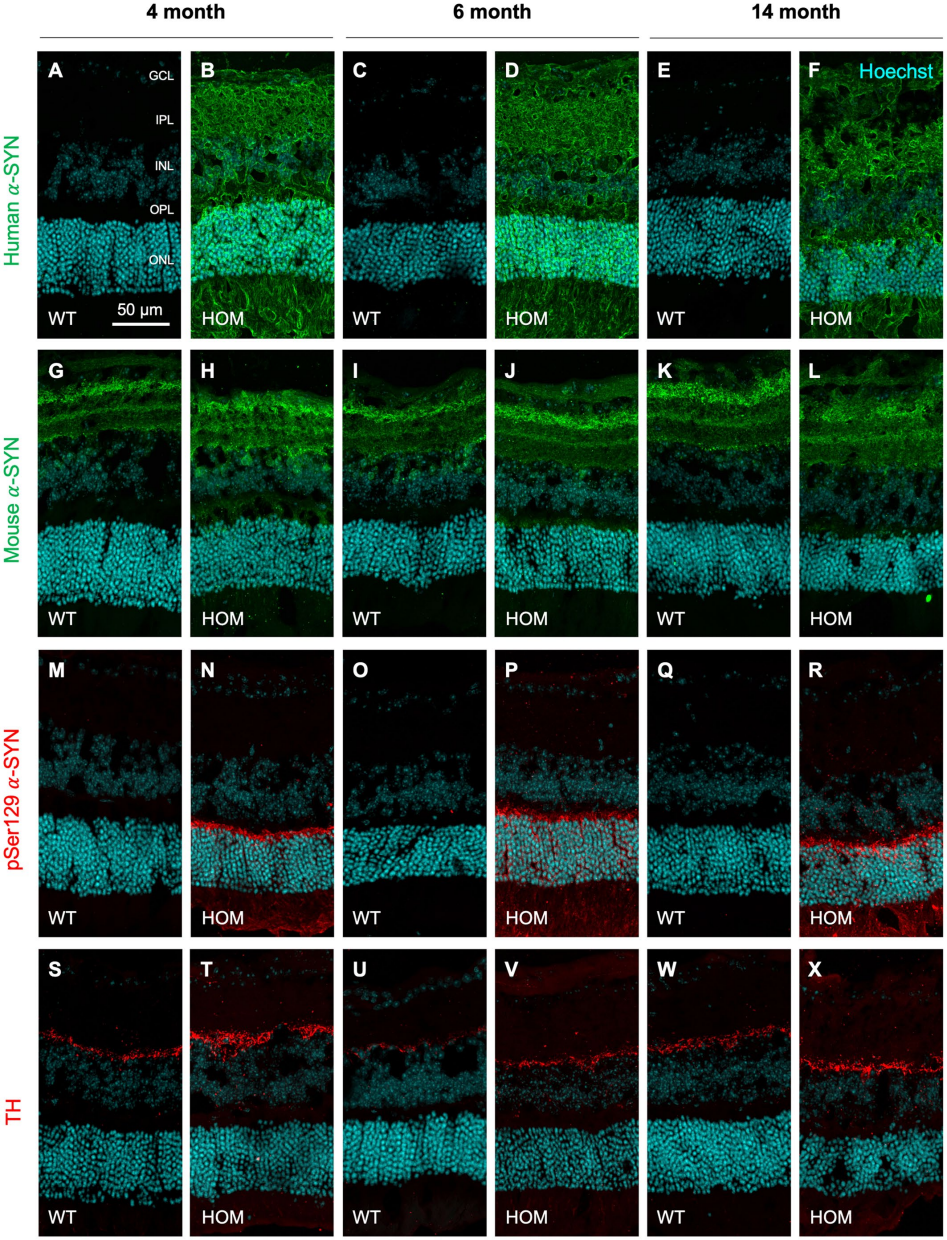
Immunohistochemical staining of alpha-synuclein and tyrosine hydroxylase in A53T retinae with age. Representative 20x confocal z-projection images of human (green, **A–F**) and mouse (green, **G–L**) alpha-synuclein (α-SYN), phosphorylated α-SYN (pSer129, red, **M–R**) and tyrosine hydroxylase (TH, red, **S–X**) staining in A53T homozygous (HOM) and wildtype (WT) control retinal cross sections at 4, 6, and 14 months of age. All retinal sections were counterstained with Hoechst cell nuclei stain as visualized in cyan. Scale bar = 50 μm. GCL, ganglion cell layer; IPL, inner plexiform layer; INL, inner nuclear layer; OPL, outer plexiform layer; ONL, outer nuclear layer.

Three types of alpha-synuclein (α-SYN) and tyrosine hydroxylase (TH) were examined in retinal cross-sections via immunohistochemistry (IHC) in A53T homozygous (HOM) and wildtype (WT) animals across 4, 6, and 14 months of age. Confirming the A53T mouse model genotype, only HOM mice were immunopositive for human α-SYN (Figures 1B,D,F) which was found throughout the retina. Furthermore, phosphorylated pSer129 α-SYN was only found in HOM retinae (Figures 1N,P,R) and was localized to the outer nuclear layer (ONL), outer margin of the outer plexiform layer (OPL) and photoreceptoral segments. Channel intensity quantification showed A53T HOM retinae had higher levels of immunopositive staining for human and phosphorylated pSer129 α-SYN than WT retinae (two-way ANOVA, genotype effect, *p* ≤ 0.0006 to 0.0001).

All groups showed staining for mouse α-SYN (Figures 1G–L) and tyrosine hydroxylase (TH, Figures 1S–X) that was localized to the inner retina (retinal nerve fiber layer, RNFL; ganglion cell inner plexiform layer, GCIPL) and inner border of the inner nuclear layer (INL), respectively. However, IHC quantification did not detect a significant difference in mouse α-SYN or TH staining between mouse strains (two-way ANOVA, genotype effect, *p* = 0.8143 to 0.5048).

#### 3.1.2. Increased Alpha-synuclein and tyrosine hydroxylase protein expression found in A53T mice

**Figure 2:**
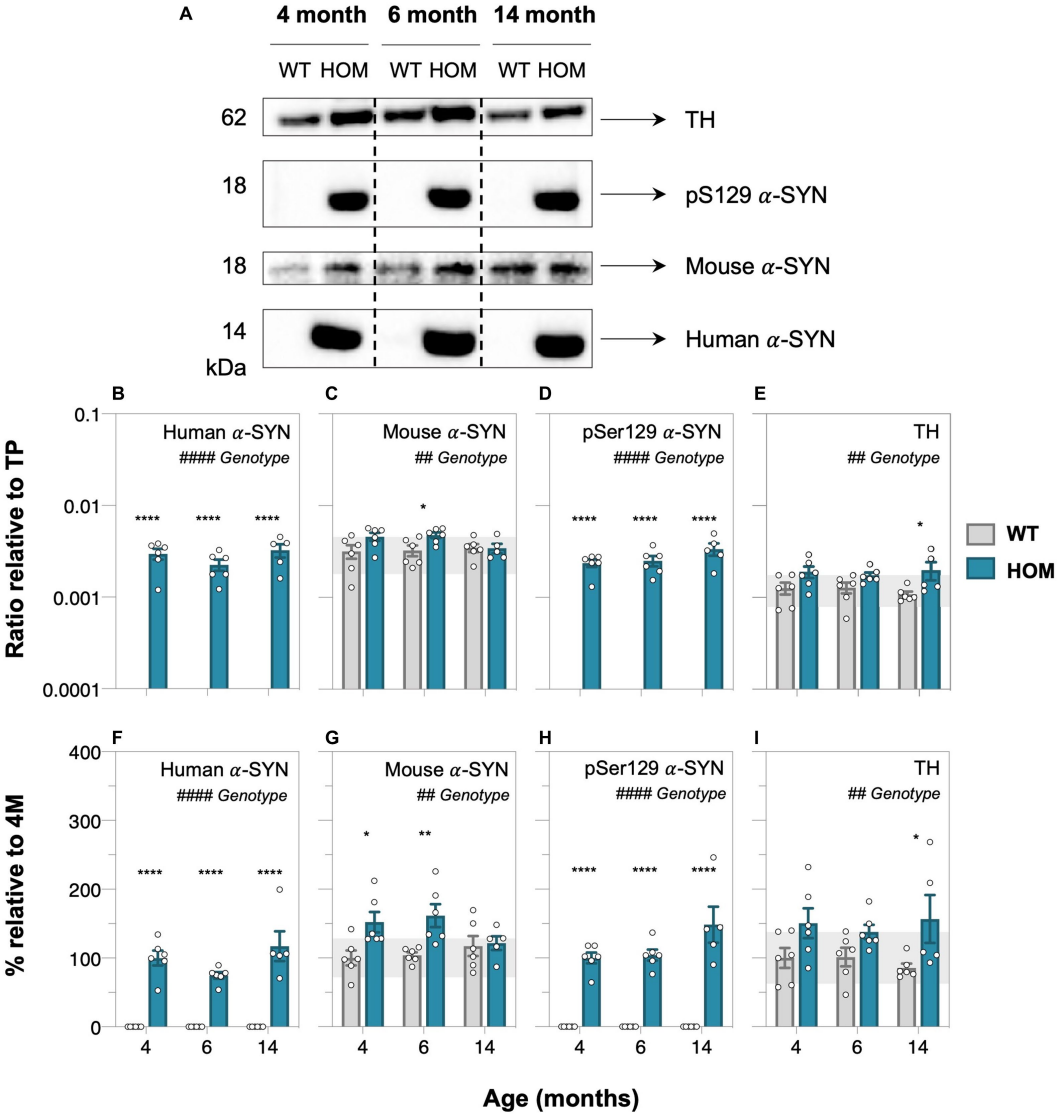
Protein abundance assessment of alpha-synuclein and tyrosine hydroxylase in A53T animals with age. **(A)** Representative western blots of retinal cell lysate from 4-, 6-, and 14-month-old A53T homozygous (HOM, *n* = 6) and wildtype (WT) littermate controls (*n* = 6) immunoblotted for tyrosine hydroxylase (TH), phosphorylated (pSer129), mouse and human alpha-synuclein (α-SYN). Quantification of western blot densitometry normalized to automated total protein measurement via ChemiDoc stain-free detection software **(B–E)** and presented as percentage (%) relative to 4-month-old (4 M) HOM group **(F,H)** or WT control **(G,I)**. A53T HOM animals had significantly increased retinal levels of all α-SYN and TH. Gray bars denote wildtype (WT) controls and teal bars denote A53T homozygous (HOM) mice. All data shown, mean ± SEM; gray shaded area, 95% CI for 4-month-old WT; ^#^*p* < 0.05 for treatment effect on two-way ANOVA analyses; *p < 0.05 for Sidak’s *post hoc* tests.

Western blot protein assessment revealed increased levels of human α-SYN (two-way ANOVA, genotype effect, *p* < 0.0001, Figure 2B; all ages, *post hoc* comparison, *p* < 0.0001), mouse α-SYN (two-way ANOVA, genotype effect, *p* = 0.0097, Figure 2C; at 4 and 6 months, *post hoc* comparison, *p* = 0.0469 to 0.0089), phosphorylated pSer129 α-SYN (two-way ANOVA, genotype effect, *p* < 0.0001, Figure 2D; all ages, *post hoc* comparison, *p* < 0.0001) and tyrosine hydroxylase (two-way ANOVA, genotype effect, *p* = 0.0012, Figure 2E; especially at 14 months, *post hoc* comparison, *p* = 0.0335) in HOM animals compared to WT controls.

### 3.2. *In vivo* retinal structure in A53T mice

#### 3.2.1. Optical coherence tomography reveals outer retinal thinning in A53T mice

**Figure 3:**
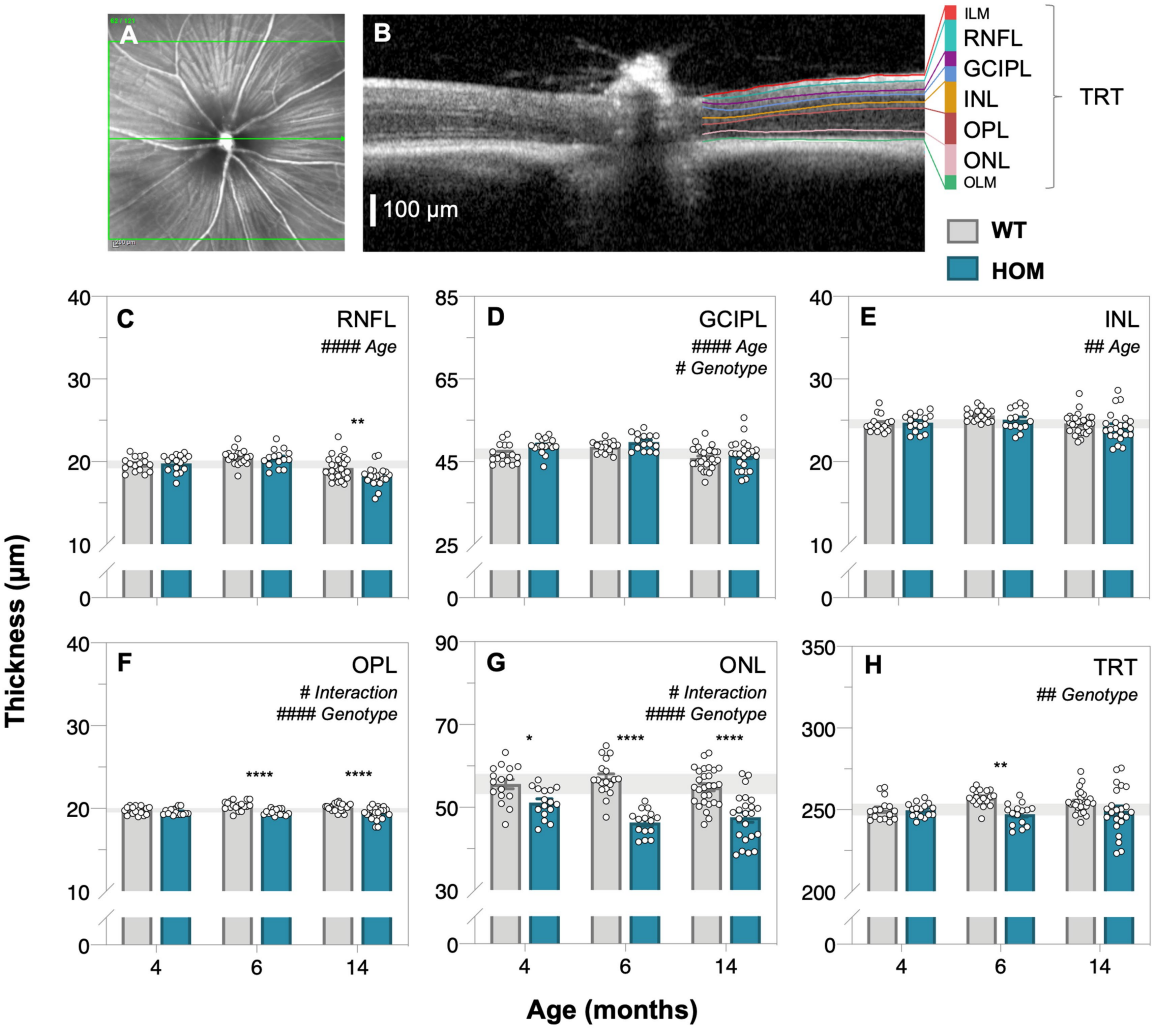
Age-related changes in retinal structure in A53T animals assessed by optical coherence tomography. Representative *en face* mouse fundus image **(A)** centered on the optic nerve head with the green line corresponding to the cross section B-scan **(B)** on the right illustrating the automatic segmentation of retinal layers; scale bar, 100 μm. **(C–H)** Raw retinal thickness values of the: retinal nerve fiber layer, RNFL; ganglion cell inner plexiform layer, GCIPL; inner nuclear layer, INL; outer plexiform layer, OPL; outer nuclear layer, ONL and total retinal thickness (TRT), respectively. TRT is a measurement that spans from the inner limiting membrane (ILM) to the outer limiting membrane (OLM). A53T HOM mice have thicker GCIPL and thinner OPL, ONL and TRT compared to WT controls. Gray bars denote wildtype (WT; 4-month, *n* = 16; 6-month, *n* = 18; 14-month, *n* = 28) controls and teal bars denote A53T homozygous (HOM; 4-month, *n* = 16; 6-month, *n* = 15; 14-month, *n* = 23) mice. All data shown, mean ± SEM; gray shaded area, 95% CI for 4-month-old WT; ^#^*p* < 0.05 for treatment effect on two-way ANOVA analyses; *p < 0.05 for Sidak’s *post hoc* tests.

Optical coherence tomography (OCT) retinal imaging was conducted to compare *in vivo* retinal structure between A53T HOM and WT control mice. Figure 3 shows a representative schematic of the segmentation of retinal layers and group averaged layer thicknesses of all animals at 4, 6, and 14 months of age. We found that A53T HOM animals had significantly thinner retinas (TRT, two-way ANOVA, genotype effect, *p* = 0.0014, Figure 3H; especially at 6 months, *post hoc* comparison, *p* = 0.0023). Significant age and genotype interactions were noted for the outer plexiform layer (OPL, two-way ANOVA, genotype effect, *p* < 0.0001, Figure 3F) and outer nuclear layer (ONL, two-way ANOVA, genotype effect, *p* < 0.0001, Figure 3G) with HOMs showing greater thinning over time compared to WT controls, starting as early as 4 months of age (Figure 3G, *post hoc* comparison, *p* = 0.018) and advancing with age (Figures 3G,H, *post hoc* comparison, *p* < 0.0001). HOM mice had slightly thicker ganglion cell inner plexiform layers (GCIPL, two-way ANOVA, genotype effect, *p* = 0.0131, Figure 3D) compared to WT controls. In contrast, the retinal nerve fiber layer (RNFL) and inner nuclear layer (INL) seemed to overall thin with age (two-way ANOVA, age effect, *p* ≤ 0.0001 to 0.0068, Figures 3C–E).

### 3.3. Retinal function in A53T mice

#### 3.3.1. Electroretinography shows impaired photoreceptor and bipolar cell function in A53T mice

**Figure 4:**
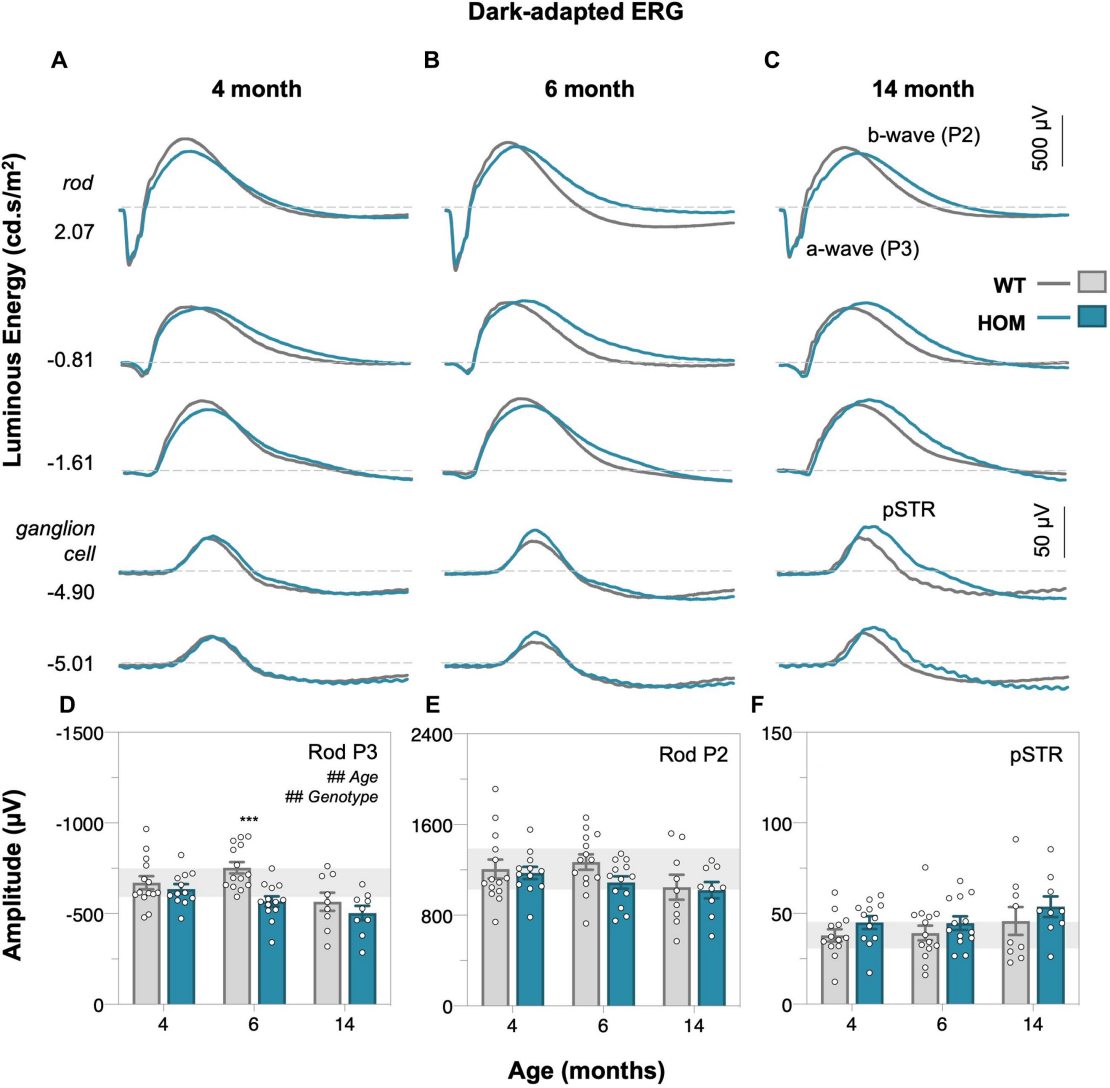
Age-related changes in dark-adapted rod retinal function of A53T mice. Group averaged dark-adapted electroretinogram (ERG) waveforms of **(A)** 4-month-old, **(B)** 6-month-old, and **(C)** 14-month-old mice. Lowest two panels show the ganglion cell dominated scotopic threshold response (STR) response (−5.01 to −4.90 log cd·s/m^2^); scale bar, 50 μV. Rod driven responses are elicited with increasing luminous energies; scale bar, 500 μV. pSTR, positive scotopic threshold response ganglion cell dominant; a-wave/P3 – photoreceptoral response; b-wave/P2 – bipolar cell response. Raw rod P3 **(D)**, rod P2 **(E)**, and pSTR **(F)** amplitudes. A53T HOM animals have decreased scoptic photoreceptoral responses. Gray traces/bars denote wildtype (WT; 4-month, *n* = 14; 6-month, *n* = 14; 14-month, *n* = 9) controls and teal traces/bars denote A53T homozygous (HOM; 4-month, *n* = 12; 6-month, *n* = 13; 14-month, *n* = 9) mice. All data shown, mean ± SEM; ^#^*p* < 0.05 for treatment effect on two-way ANOVA analyses; *p < 0.05 for Sidak’s *post hoc* tests; gray shaded area, 95% CI for 4-month-old WT.

**Figure 5:**
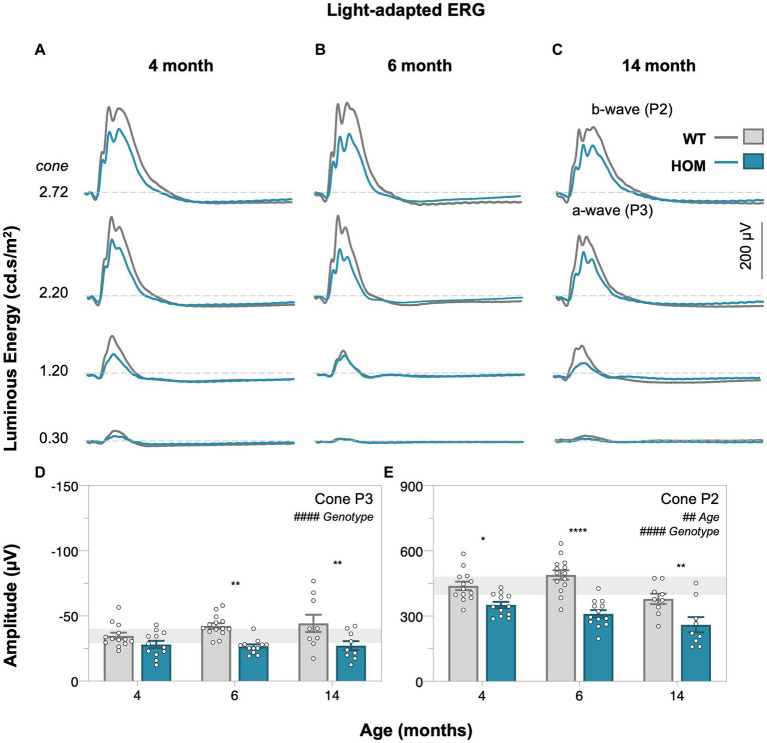
Age-related changes in light-adapted cone retinal function of A53T mice. Group averaged light-adapted electroretinogram (ERG) waveforms of **(A)** 4-month-old, **(B)** 6-month-old, and **(C)** 14-month-old mice; scale bar, 200 μV. a-wave/P3 – photoreceptoral response; b-wave/P2 – bipolar cell response. Raw cone P3 **(D)** and cone P2 **(E)** amplitudes of A53T animals. A53T HOM animals have decreased photopic photoreceptoral and cone bipolar cell responses. Gray traces/bars denote wildtype (WT; 4-month, *n* = 13; 6-month, *n* = 14; 14-month, *n* = 9) controls and teal traces/bars denote A53T homozygous (HOM; 4-month, *n* = 12; 6-month, *n* = 13; 14-month, *n* = 9) mice. All data shown, mean ± SEM; ^#^*p* < 0.05 for treatment effect on two-way ANOVA analyses; *p < 0.05 for Sidak’s *post hoc* tests; gray shaded area, 95% CI for 4-month-old WT.

Full field electroretinography (ERG) was used to compare retinal function between A53T HOM and WT control animals. Dark-adapted (Figure 4, rod-dominated) and light adapted (Figure 5, cone only) ERG responses were measured. At dim light levels (−5.01 to −4.90 log cd·s/m^2^), the ERG waveform is dominated by the scotopic threshold response (STR, lower panels of Figures 4A–C). With brighter luminous energies, the electronegative photoreceptoral a-wave followed by a large bipolar cell b-wave became increasingly evident (upper panels of Figures 4A–C). Light adaptation enabled the isolation of cone-dominated ERG responses (Figures 5A–C).

We found that A53T mice had attenuated rod and cone P3 (two-way ANOVA, genotype effect, *p* = 0.0019 and *p* < 0.0001, Figures 4D, 5D, respectively) and cone P2 (two-way ANOVA, genotype effect, *p* < 0.0001, 5E) amplitudes in comparison to WT controls. By 4 months of age there was a deficit in cone bipolar cell (P2) function (*post hoc* comparison, genotype effect, *p* = 0.0117, Figure 5E), although there was a similar magnitude in cone photoreceptor (P3) function at this age, greater variability in this parameter made it harder to find a *post hoc* difference between strains. In general, rod P3 and cone P2 responses appeared to decrease with age (two-way ANOVA, age effect, *p* = 0.0011 to 0.0334). Inner retinal function as denoted by the positive scotopic threshold response (pSTR) was conserved across all ages (two-way ANOVA, genotype effect, *p* = 0.0728, Figure 4F).

#### 3.3.2. Outer retinal thinning correlates with impaired retinal function in A53T mice

**Figure 6:**
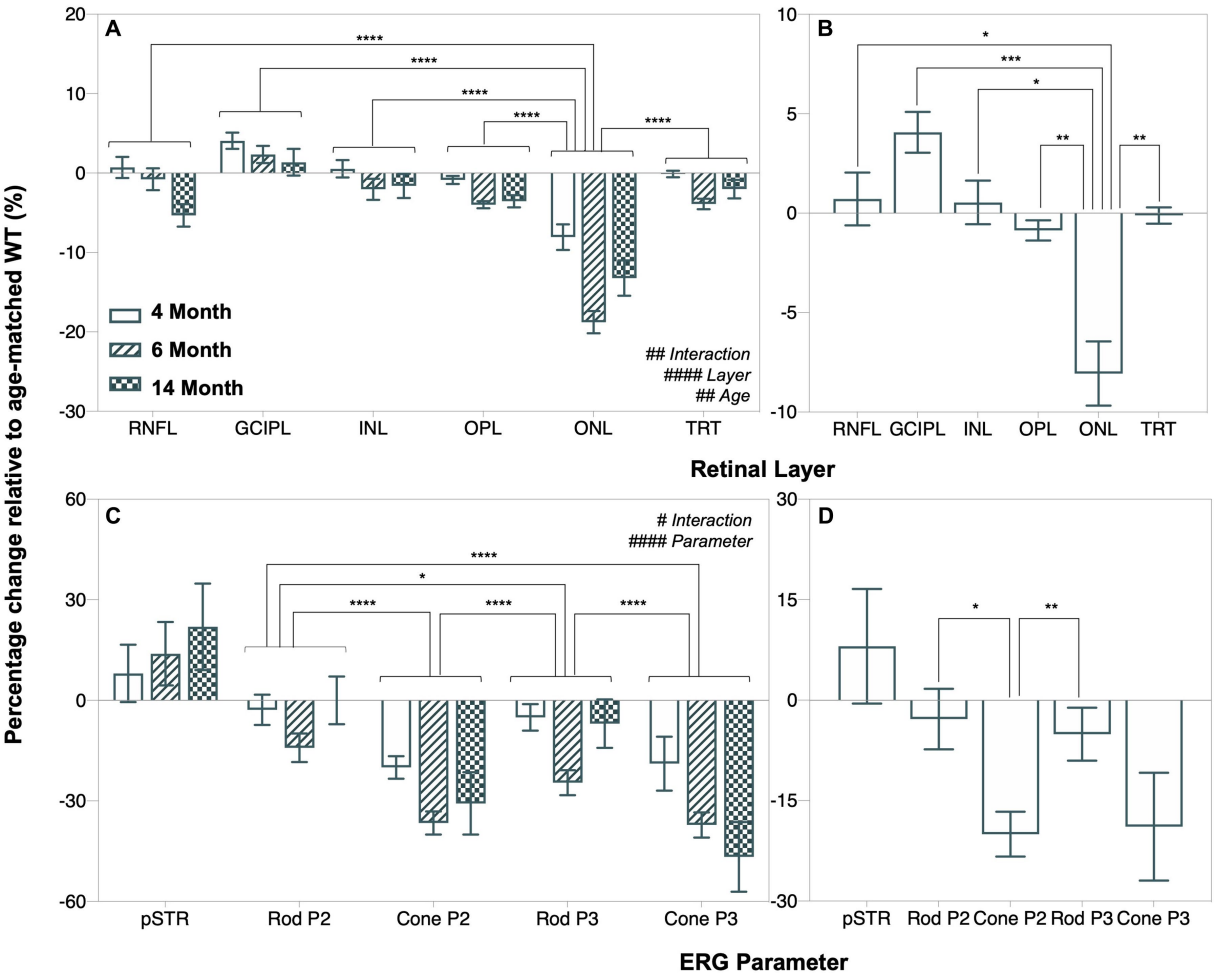
Age-related changes in retinal function and structure in A53T animals expressed relative to age-matched wildtype controls. **(A,B)** Retinal layer thicknesses (retinal nerve fiber layer, RNFL; ganglion cell inner plexiform layer, GCIPL; inner nuclear layer, INL; outer plexiform layer, OPL; outer nuclear layer, ONL and total retinal thickness, TRT) expressed as a percentage change relative to age-matched wildtype (WT) A53T animals. **(C,D)** Percentage change of ERG parameters (pSTR, rod P2, cone P2, rod P3 and cone P3 amplitudes) relative to age-matched wildtype A53T animals. ONL thinning and reduced cone P2 amplitude were the most salient and earliest retinal changes in A53T HOM animals. *Post hoc* analysis also showed that the pattern of change in the positive scotopic threshold response (pSTR) was significantly different from all other ERG parameters, but this is not shown in the figure for simplicity. Wildtype (WT; 4-month, *n* = 14–16; 6-month, *n* = 14–18; 14-month, *n* = 8–28) control littermates versus A53T homozygous (HOM; 4-month, *n* = 12–16; 6-month, *n* = 13–15; 14-month, *n* = 9–19) mice. All data shown, mean ± SEM.

As shown in Figure 6A, outer retinal thinning (OPL and ONL) precedes inner retinal thinning (RNFL, exclusively at 14 months, *post hoc* comparison, *p* = 0.0079, Figure 3C) in A53T HOM animals as indicated by retinal thickness changes expressed as a percentage relative to respective WT controls. ONL thinning was the most salient structural change in A53T HOM animals and occurred as early as 4 months of age (Figure 6B). Figure 6C compares the magnitude of deficits in various ERG components by expressing individual values relative to their respective age-matched WT control. It shows the largest relative deficit in retinal function lay within cone driven ERG responses, specifically in the cone P2 bipolar cell response which was already evident at 4 months of age (Figure 6D).

**Figure 7:**
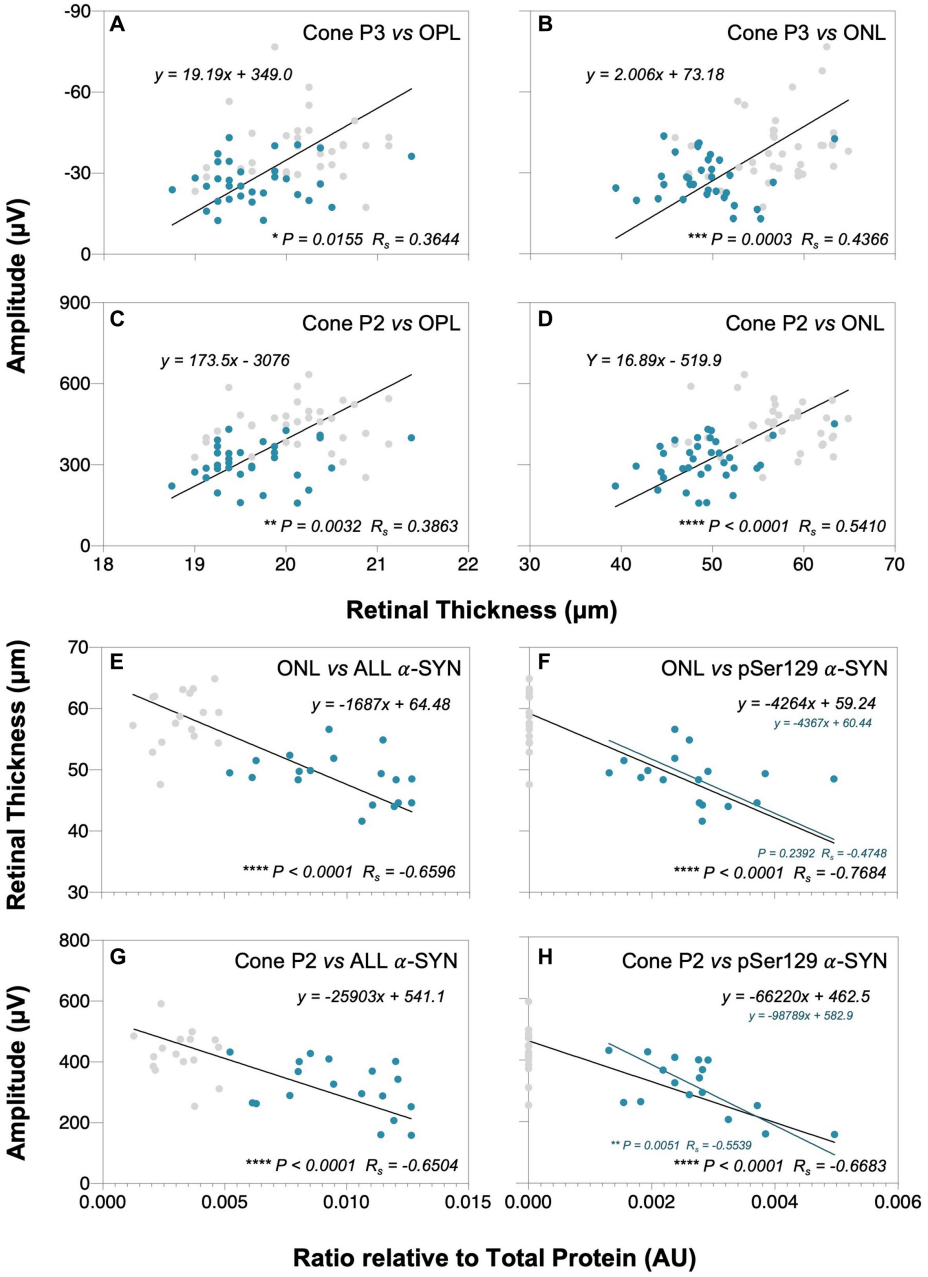
Correlations between retinal α-SYN, structure and function. Deming linear regression analysis of outer plexiform layer (OPL) and outer nuclear layer (ONL) thicknesses with cone P3 **(A,B)** and cone P2 **(C,D)** amplitudes, and total alpha-synuclein (addition of mouse α-SYN, human α-SYN and phosphorylated α-SYN, denoted as ALL) and phosphorylated α-SYN (pSer129 α-SYN) levels with ONL thickness **(E,F)** and cone P2 amplitude **(G,H)**, respectively. Modest correlations were found between outer retinal layer thickness and cone ERG parameters and between total and pSer129 α-SYN levels in A53T animals that correlated with ONL thinning and reduced cone P2 amplitudes. Gray dots denote wildtype (WT; *n* = 18–37) controls and teal dots denote A53T homozygous (HOM; *n* = 18–34) mice. Spearman’s R (R_s_) is given.

As shown by Deming linear regression analyses in Figure 7, modest correlations between outer retinal layer thickness, ERG parameters and retinal α-SYN levels were found. In particular, the strongest structure–function correlation lay between outer nuclear layer (ONL) thickness and cone P2 (*p* < 0.0001, R_s_ = 0.5410, Figure 7D) amplitude. Furthermore, we found negative correlations between total retinal α-SYN and phosphorylated (pSer129) α-SYN levels and ONL thickness (*p* < 0.0001, R_s_ = −0.6596, Figure 7E and *p* < 0.0001, R_s_ = −0.7684, Figure 7F, respectively) and cone P2 amplitude (*p* < 0.0001, R_s_ = −0.6504, Figure 7G and *p* < 0.0001, R_s_ = −0.6683, Figure 7H, respectively).

## 4. Discussion

Our study is the first to investigate *in vivo* retinal structure and function changes with advancing age in the A53T mouse model of PD. We find that the accumulation of phosphorylated alpha-synuclein in the outer nuclear layer of homozygous animals correlates with neurodegenerative thinning of this area that is associated with attenuated retinal function, especially in cone responses.

### 4.1. Proteinaceous and dopaminergic PD pathological hallmarks found in A53T mouse retina

Our findings of elevated levels of α-SYN and tyrosine hydroxylase in A53T retinal tissue *via* immunohistochemical staining in combination with western blotting provide robust evidence that aberrant protein formation and dopaminergic dysfunction occur in the eyes of A53T homozygous mice. This is consistent with elevated levels of α-SYN and dopamine active transporter (DAT) seen in the brains of A53T mice (Oaks et al., 2013).

We show across all ages that human α-SYN (Figures 1B,D,F) is diffusely localized throughout the retina of HOM mice whereas the phosphorylated (pSer129) toxic form of human α-SYN (Figures 1N,P,R) specifically deposits in and around the outer nuclear layer (ONL). This is in agreement with, Mammadova et al. (2019) who found that in A53T mice aged 5 to 18 months old, pSer129 was largely localized to the outer nuclear layer whereas total α-SYN (BD Biosciences, 610,786) was widespread across the retina. In saying this, the pattern of pSer129 α-SYN deposition seen in A53T mice differs to those established in other PD animal models such as the Thy-1 A30P mouse and human patients where pSer129 α-SYN has been localized to the inner retina (Veys et al., 2019, 2021), particularly in the ganglion cell layer (GCL) and inner plexiform layer (IPL). This is most likely due to the models’ intrinsic genetic promoters (i.e., prion protein [PrP] versus Thy-1) which are known to differentially affect expression levels and phenotypic translation (Zhang et al., 2022). As such, care must be taken when considering findings from the A53T model as the method of pSer129 α-SYN accumulation in mice and PD patients may vary.

In addition, we establish that HOM animals have increased levels of mouse α-SYN (localized to the ganglion cell inner plexiform layer [GCIPL], Figures 1G–L) compared to controls, particularly at the younger ages of 4 and 6 months old (Figures 2C,G). ELISA quantification of mouse α-SYN using the D37A6 antibody in the brain by Bétemps et al. (2014) showed distinct immunoreactivity patterns in pathological brain regions of sick and inoculated M83 mice. This data in combination with our findings suggest that the recruitment of mouse α-SYN may contribute to the aggregation of pathological α-SYN, particularly at early stages of disease progression.

We also found that in comparison to WT controls, A53T HOM mice have elevated tyrosine hydroxylase levels (Figures 1S–X, 2E,I), especially at 14 months of age. Marrocco et al. (2020) show a significant loss of TH positive dopaminergic A18 amacrine cells occurs in an adeno-associated viral (AAV) induced mouse model of α-SYN overexpression. It is known that toxic α-SYN oligomers are stabilized by dopamine (Conway et al., 2001) and thought that intracellular dopamine levels may increase with abnormal α-SYN (Lotharius and Brundin, 2002). As such, the overaccumulation of α-SYN in the retina may explain the selective vulnerability of dopaminergic amacrine cells within these α-SYN mouse models (Finkelstein et al., 2016; Costa et al., 2020). Our finding of increased TH levels in A53T HOM mice may speak to possible compensatory mechanisms in surviving dopaminergic retinal neurons to increase functional activity, as shown in mouse (Kolacheva et al., 2022) and human (Mogi et al., 1988) studies of the Parkinsonian brain. This finding is also in agreement with previous findings in the A53T model in the brain with increases in other dopamine regulators such as DAT (Oaks et al., 2013).

### 4.2. Outer retinal structure most affected in A53T mice

To our knowledge, this is the first study to quantify changes in retinal thickness in the A53T mouse model of PD. We find that, as early as 4 months of age OPL and ONL (Figures 3F,G) thinning occur in A53T HOM mice. Clinical human PD literature predominantly report thinning of inner retinal layers and total retinal thickness (Huang et al., 2020, 2021; Zhou et al., 2021). The current study also finds thinning of the RNFL and TRT akin to previous human studies (Figures 3C,H), at the later 6 and 14 months of age.

Only a few groups have examined outer retinal thickness in human PD patients (Garcia-Martin et al., 2014; Müller et al., 2014; Roth et al., 2014; Chorostecki et al., 2015). Significant ONL thinning have been established in multiple PD patient cohorts compared to age-matched controls (Albrecht et al., 2012; Roth et al., 2014; Schneider et al., 2014; Unlu et al., 2018). OPL changes in PD are less consistent, with Chorostecki et al. (2015) reporting a significant increase in OPL volume and Garcia-Martin et al. (2014) finding a decrease in OPL thickness. Whether these differences are due to slight dissimilarity in disease severity or age between cohorts (Hoehn Yahr score: 2 vs. 2.7 and disease duration: mean 6.4 years vs. median 8.4 years, respectively) requires further investigation. It is also important to note that it is difficult to accurately measure the OPL and ONL in macular OCT scans due to orthogonal convergence of Henle Fibers (Lujan et al., 2011) at the fovea and thus this may contribute to the variability.

With advancing age, we show more generalized retinal thinning in A53T mice, with significant reductions in the inner retinal layers including the RNFL, GCIPL and INL especially at 14 months that follow outer retinal thinning (Figure 6A). In addition, we discovered modest thickening in the GCIPL in HOM mice at 4 and 6 months compared to WT controls (Figure 3D) which has also previously been shown in Parkin−/− mice (Hu et al., 2022). Possible causes of the GCIPL thickening include impaired mitochondria as per the Parkin−/− mice (Hu et al., 2022), gliosis or local accumulation of mouse α-SYN to the inner retina. In this study the correlation between mouse α-SYN and GCIPL was not significant (Supplementary Figure 1C) indicating that perhaps another mechanism may be at play.

### 4.3. Photoreceptor and downstream bipolar cell dysfunction occurs in A53T mice

With respect to retinal function, we show for the first time using electroretinography (ERG) that significant attenuation in dark-adapted (Figure 4) and light-adapted (Figure 5) photoreceptoral and bipolar cell responses occur in A53T HOM animals. The reduction in bipolar cell function, being of a similar percentage (Figure 6C) is likely to be driven by a loss of upstream photoreceptor function (Perlman, 1983; Nguyen et al., 2013). Interestingly the light adapted cone pathway showed greater and earlier dysfunction than the dark-adapted rod pathway (Figures 6C,D). A similar preferential loss of cone pathways was noted in a mouse model of intravitreal injection of adeno-associated viral (AAV) vector to induce overexpression of human α-SYN (Marrocco et al., 2020).

These ERG deficits in A53T mice are largely consistent with reports of photoreceptoral and bipolar cell dysfunction as measured by full-field flash ERG in clinical PD cohorts (Gottlob et al., 1987; Burguera et al., 1990; Devos et al., 2005; Nowacka et al., 2015; Mello et al., 2022). Within clinical studies a direct comparison between rod and cone ERG pathways has not been directly examined. Although the downstream deficit in bipolar cell amplitude mirrored that of photoreceptors, this was not the case for downstream retinal ganglion cell function. A preservation of the ganglion cell response has previously been reported in ageing studies (Charng et al., 2011) and is thought to represent a compensatory mechanism to maintain output to the brain. Further studies are required to determine whether this is the case in the A53T mouse.

### 4.4. Retinal structure and function correlates with alpha-synuclein levels

As photoreceptors are the first cell class in the retinal pathway and their *in vivo* integrity can be measured with ONL and OPL thickness on OCT as well as P3 and downstream P2 changes with ERG these parameters were correlated. In this manner, significant relationships (*p* < 0.0001) were found between outer retinal structure (ONL and OPL) and functional cone photoreceptor and bipolar cell responses (Figure 7).

One of the advantages of conducting animal studies is that tissue can be assayed, and mechanistic insights inferred. As such retinal levels of the toxic form of α-SYN (pSer129) and an overall measure of alpha-synuclein (pSer129, human α-SYN, mouse α-SYN) were correlated again the most salient retinal structure (ONL) and function changes (cone P2) from Figure 6. Here we found that higher levels of α-SYN proteins (pSer129, human α-SYN, mouse α-SYN), resulted in thinner ONL and poorer cone P2 function (Figures 7E,G). This relationship was also evident for pSer129 α-SYN which was localized to the ONL (Figures 7F,H). Correlations between the downstream cone P3 and retinal α-SYN levels (total and pSer129) also trended towards similar relationships but did not reach significance, possibly due to higher variability in the cone P3 parameter (Supplementary Figures 1A,B).

In summary, the A53T model of PD characterized here recapitulates retinal hallmarks found in human PD including loss of photoreceptoral ERG a-wave, corresponding structural thinning in the ONL and α-SYN deposition in the retina. Although current human tissue examinations have indicated that pSer129 α-SYN is largely deposited in the inner retina of PD patients, the characterization of and correlation between retinal α-SYN levels versus function and structure in A53T mice aides our progression in this field as it contributes to our understanding of the different ways α-SYN accumulation may impact the retina in PD.

## 5. Conclusion

To our knowledge, this is the first study to characterize the retinal phenotype of A53T mice at ages corresponding to early, mid, and advanced stages of PD pathology. We show that the overexpression of phosphorylated human α-SYN in A53T mice preferentially affects the outer retina and manifests as neurodegenerative thinning in the outer plexiform and nuclear layers that is associated with reductions in rod and more so cone photoreceptoral and bipolar cell function. These changes occur as early as 4 months of age, suggesting they may be useful early biomarkers of α-SYN associated pathology. As such, these findings provide us with a deeper understanding of the pattern and utility of alpha-synuclein driven changes that may occur in the retina with Parkinson’s disease progression.

## Data availability statement

The raw data supporting the conclusions of this article will be made available by the authors, without undue reservation.

## Ethics statement

The animal study was reviewed and approved by the Florey Institute of Neuroscience and Mental Health Animal Ethics Committee (Approval number: 17-046-UM).

## Author contributions

CN, DF, BB, and KT conceptualized the study, designed the experiments, and wrote the manuscript. KT, VW, AH, and CN collected and analyzed the data. VW and AH reviewed the manuscript. All authors contributed to the article and approved the submitted version.

## Funding

This research was funded by U.S. Army Medical Research Acquisition Activity, 820 Chandler Street, Fort Detrick MD 21702-5014 (US Department of Defense, CDMRP PD210055) and Australian Research Council Linkage grants (LP160100126), as well as funding through Melbourne Neuroscience Institute Interdisciplinary Seed Fund, Melbourne Research Fellowship, Melbourne Neuroscience Institute Fellowship, Melbourne School of Health Sciences Seed Funding and John Landman PhD Scholarship.

## Conflict of interest

CN and BB are joint investigators on an Australian Research Council Linkage grant LP160100126 with AstraZeneca Neuroscience and Biogen Inc.

The remaining authors declare that the research was conducted in the absence of any commercial or financial relationships that could be construed as a potential conflict of interest.

## Publisher’s note

All claims expressed in this article are solely those of the authors and do not necessarily represent those of their affiliated organizations, or those of the publisher, the editors and the reviewers. Any product that may be evaluated in this article, or claim that may be made by its manufacturer, is not guaranteed or endorsed by the publisher.
