# Supplementary material for: Retinal alpha-synuclein accumulation correlates with retinal dysfunction and structural thinning in the A53T mouse model of Parkinson’s disease
Source: Front Neurosci. 2023 May 5;17:1146979. doi: 10.3389/fnins.2023.1146979 (PMC10196133; doi:10.3389/fnins.2023.1146979)
Supplement: Supplementary file 1 [file Data_Sheet_1.docx]

Supplementary Material

Retinal Alpha-synuclein Accumulation Parallels Retinal Dysfunction & Structural Thinning in the A53T Mouse Model of Parkinson’s Disease

Katie K.N. Tran^1^, Vickie H.Y. Wong^1^, Anh Hoang^1^, David I. Finkelstein^2^, Bang V. Bui^1^, Christine T.O. Nguyen^1*^

^1^Ocular Biomarkers Laboratory, Department of Optometry and Vision Sciences, The University of Melbourne, Parkville, Victoria, Australia.

^2^Parkinson’s Disease Laboratory, The Florey Institute of Neuroscience and Mental Health, The University of Melbourne, Parkville, Victoria, Australia.

*** Correspondence:** Christine T.O. Nguyen: [christine.nguyen@unimelb.edu.au](mailto:christine.nguyen@unimelb.edu.au)

# Supplementary Figures and Tables

## Supplementary Figures


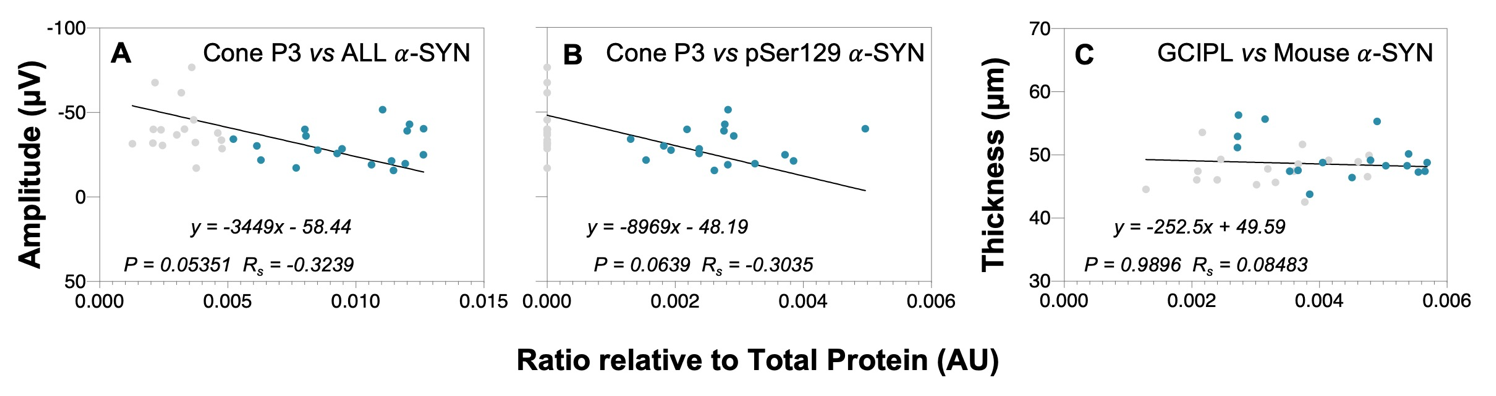


**Supplementary Figure 1.** **Additional correlations between retinal α-SYN, structure and function.** Deming linear regression analysis of total alpha-synuclein (addition of mouse α-SYN, human α-SYN and phosphorylated α-SYN, denoted as ALL) and phosphorylated α-SYN (pSer129 α-SYN) levels with cone P3 amplitude (**A**, **B**) and ganglion cell inner plexiform layer (GCIPL) thickness with mouse α-SYN levels (**C**), respectively. Gray dots denote wildtype (WT; *n* = 18-37) controls and teal dots denote A53T homozygous (HOM; *n* = 18-34) mice. Spearman’s R (R_s_) is given.

**
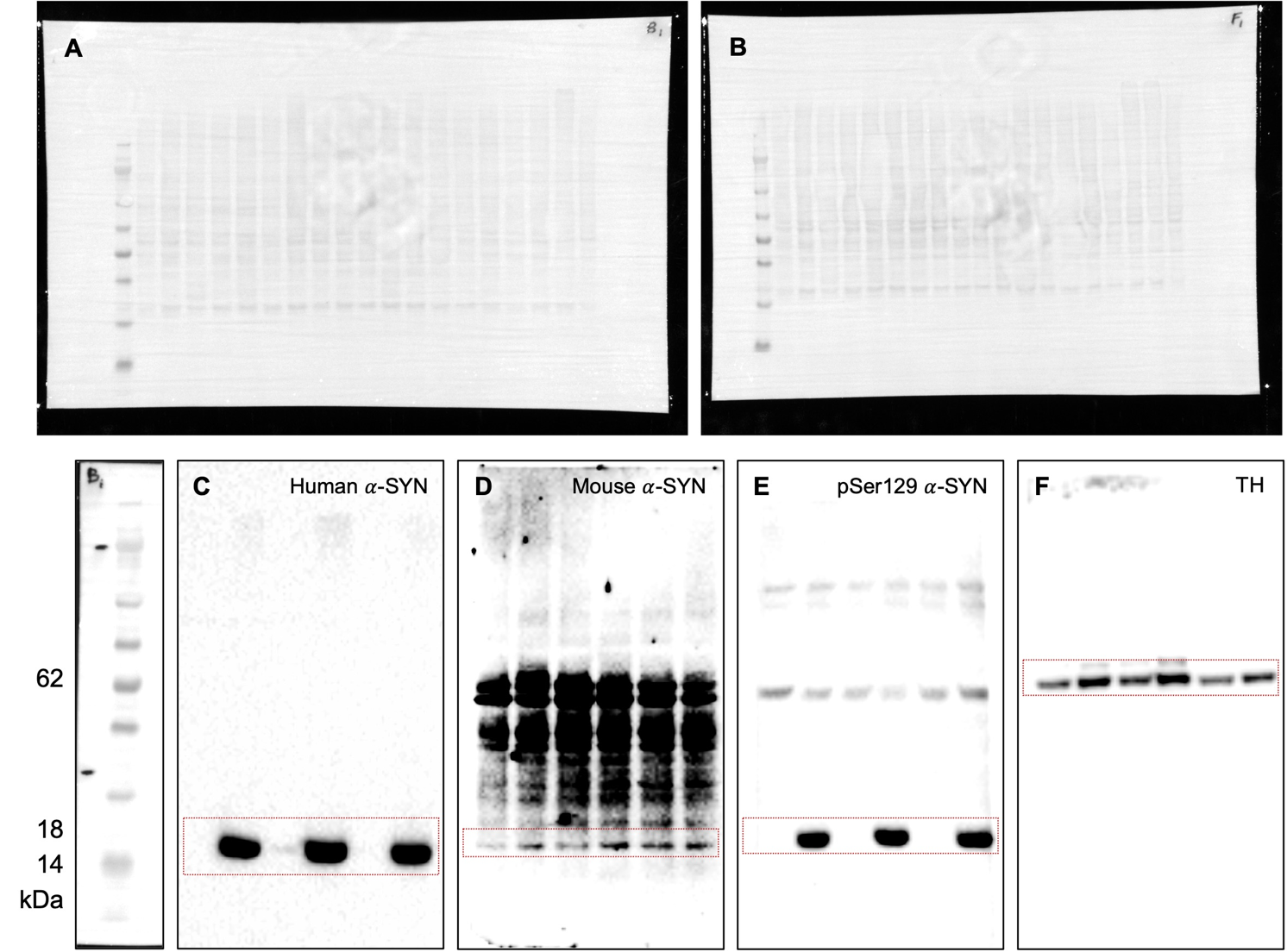
**

**Supplementary Figure 2.** **Western Blots of A53T and WT retinae.** All quantitative Western blot data and analysis was obtained from two individual retinal sample (*n* = 6) blots (**A-B**) which were probed with human α-SYN (**C**), mouse α-SYN (**D**), phosphorylated (pSer129) α-SYN (**E**) and tyrosine hydroxylase (TH, **F**) as indicated by the red dotted rectangle on the provided representative blots (pooled *n* = 6 retinae per lane).
